# Surface Chemical Changes of Sugar Maple Wood Induced by Thermo-Hygromechanical (THM) Treatment

**DOI:** 10.3390/ma12121946

**Published:** 2019-06-17

**Authors:** Qilan Fu, Alain Cloutier, Aziz Laghdir, Tatjana Stevanovic

**Affiliations:** 1 Center de Recherche sur les Matériaux Renouvelables (Renewable Materials Research Center), Département des Sciences du bois et de la Forêt (Department of Wood and Forest Sciences), Université Laval, Québec, QC G1V 0A6, Canada; qilan.fu.1@ulaval.ca (Q.F.); Tatjana.Stevanovic@sbf.ulaval.ca (T.S.); 2 Service de Recherche et D’expertise en Transformation des Produits Forestiers (Research and Expertise Service on Processing of Forest Products), 25 Armand-Sinclair, Porte 5, Amqui, QC G5J 1K3, Canada; aziz.laghdir@serex.qc.ca

**Keywords:** wood densification, depolymerization, surface chemical composition, anhydrous sugar

## Abstract

The aim of this study was to investigate the effects of heat and steam on the chemical properties of thermo-hygromechanical (THM)-densified sugar maple wood. The THM densification process was performed at two different temperatures (180 °C and 200 °C) with and without steam. The functional groups, surface chemical composition and internal structure and components of the control and densified samples were investigated using attenuated total reflection Fourier transform infrared (ATR-FTIR), X-ray photoelectron (XPS) spectroscopy and pyrolysis gas chromatography-mass spectrometry (Py-GC/MS). The obtained results suggest that the THM densification treatment resulted in significant chemical changes on the wood surface. The results of the ATR-FTIR spectra confirmed the decomposition of hemicelluloses and the relative increase of cellulose and lignin contents on the wood surface. The Py-GC/MS and XPS results show an increase of the oxygen/carbon atomic (O/C) ratio, which indicated that chemical substances containing oxygenated functionality were formed through the densification process. The densification treatment favored the depolymerization of hemicelluloses and cellulose as indicated by an increased anhydrous sugar (levoglucosan) release during the pyrolysis process. Densification also facilitated the cleavage of the lignin side chains, resulting in increased phenyl units with short chains released during the pyrolysis process.

## 1. Introduction

The sugar maple (*Acer saccharum* Marsh.) is an important commercial hardwood in Canada, which has a relatively high hardness, dense grain and light color. This species is widely used in the manufacture of furniture, flooring, farm tools, cutting blocks, fuelwood, veneer and other products [1]. The thermo-hygromechanical (THM) densification process involves the utilization of steam, heat and pressure to compress the wood structure. The main purpose of densification is to enhance wood density, which improves its mechanical performance and commercial value. In recent years, many research efforts have been dedicated to using the appropriate densification process to improve wood properties [2,3,4,5,6,7,8,9,10]. Koumba et al. [11] reported that the wood densification process acts as a mild thermo-chemical pre-treatment which can impact biomass composition and structure.

During wood densification, particularly at a high treatment temperature, the individual wood components undergo different chemical degradations. The extent of degradation depends on many factors, such as temperature, wood moisture content and processing time. Most extractible compounds are formed by the alteration and degradation of hemicelluloses and lignin during thermal treatment [12,13]. As wood is heated, acetic acid is formed by the hydrolysis of acetylated hemicelluloses. The released acid works as a catalyst to facilitate the hydrolysis of hemicelluloses into oligomeric and monomeric structures. Subsequently, the oligomeric and monomeric sugar units will be further dehydrated to aldehydes, of which furfural is formed from pentose sugar units and hydroxymethyfurfural is formed from the dehydration of hexose sugar units [14]. Among the three major components, lignin is the least reactive. However, at high temperature, bonds between phenyl propane units can be partly cleaved and some condensation reactions may occur. According to our knowledge, diphenylmethane-type condensation is the most typical reaction at the temperature range of 120 °C to 220 °C. This reaction is probably the main cause of the darker color of wood after thermal treatment [15].

Although no chemical agents are added during THM densification [16], it is reasonable to consider that certain chemical changes occur on the densified wood surface that are responsible for its changes of color and wettability. Since wood structure and chemical composition have important effects on the physical and mechanical properties, a fundamental investigation of the changes of wood constituents is needed to understand the changes of the wood’s physical and mechanical properties occurring during the THM densification process [16].

Surface spectroscopic techniques, such as X-ray photoelectron spectroscopy (XPS), attenuated total reflection Fourier transform infrared spectroscopy (ATR-FTIR) and pyrolysis gas chromatography-mass spectrometry (Py-GC/MS), are normally used to analyze wood surface chemical compositions, functional groups and component structure. Belleville et al. [13] investigated the thermo-chemical changes of sugar maple and yellow birch occurring during wood welding using FTIR, XPS and Py-GC/MS. Their results indicated that a degradation of hemicelluloses occurred and chemical substances with oxygenated functionality were formed. Meanwhile, FTIR could confirm the cleavage of the lignin ether bonds and the release of free phenolic hydroxyls. Diouf et al. [17] found that hemicelluloses degraded at 160 °C, high carbon compounds and extractives were removed and a new lignin complex was formed at higher temperatures. Koumba et al. [18] characterized the chemical changes in the wood constituents after different densification treatments using FTIR, Py-GC/MS and XPS. Their results indicated that the anhydrous sugar content of the wood surface increased after densification, which revealed carbohydrate losses during densification.

The objective of this study was to characterize the chemical changes (functional groups and surface chemical compositions) of wood treated under diverse thermal and hygrometric conditions. This investigation can help to develop a better understanding of the THM densification process. The effects of heat and steam on the mechanical properties and dimensional stability of THM-densified wood were investigated in our previous research [16]. These results suggested that, compared to the samples densified without steam, the samples densified with steam showed higher values for hardness, bending strength, bending stiffness, compression set and density when treatment temperature was below 200 °C. A better dimensional stability was also observed. These different properties for densified wood might be caused by different extents of degradation of the wood’s main chemical compositions treated under diverse THM densification conditions. Hence, the examination of surface chemical composition changes of densified wood could also further help to explain and clarify why the mechanical properties of wood increased after densification treatment and why wood densified with steam has a better dimensional stability than that of wood densified without steam.

## 2. Materials and Methods

Thin sawn strips of sugar maple (*Acer saccharum* Marsh.) wood obtained from a hardwood flooring plant were used (Lauzon, Distinctive Hardwood Flooring Inc., Papineauville, QC, Canada). Their average apparent density (at 20 °C and 65% relative humidity (RH)) was 734 kg/m^3^ and their dimensions were 5.7 mm (radial) × 84.0 mm (tangential) × 695.0 mm (longitudinal). When they were received, the strips were stored in a conditioning room at 20 °C and 65% RH until an equilibrium moisture content of approximately 12% was achieved. Five groups of 8 strips of sugar maple wood samples (4 groups densified at 180 °C and 200 °C with and without steam, respectively, with one group of control samples) were prepared. Samples for chemical analysis (XPS and Py/GC-MS) were collected by scraping material from the wood surfaces of the control and densified samples. The scraped materials from the 8 strips per each group were well mixed and were grinded into powder. All of the testing samples were oven-dried prior to their chemical characterization.

### 2.1. Thermo-Hygromechanical Densification Process

**Figure 1:**
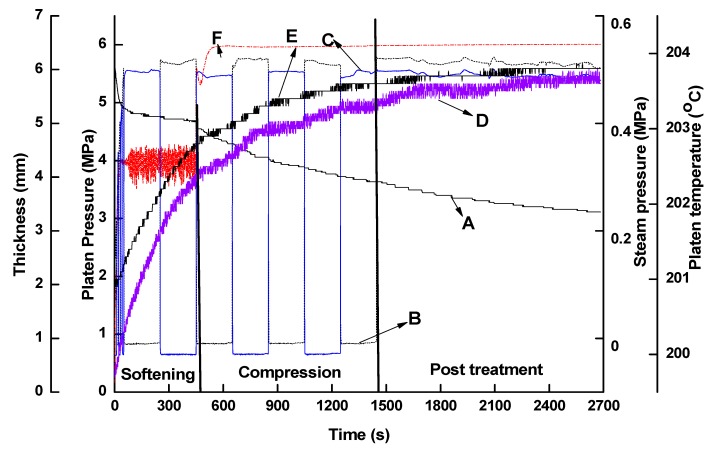
Thermo-hygromechanical densification process. A: Thickness; B, C: Top and bottom platen steam pressures, respectively; D, E: Top and bottom platen temperatures, respectively; F: Platen load.

The THM densification process with and without steam injection was previously presented by Fu et al. [16]. A steam injection press (Dieffenbacher, Alpharetta, GA, USA) with dimensions of 862 mm × 862 mm was used for the densification treatment. The two platens were preheated to the target temperature before treatment. The upper platen reached the specimens within 86 s. The whole densification process (Figure 1, Fu et al. [9]) can be divided into three steps: Wood softening (duration of 400 s), compression (duration of 1000 s) and post-treatment (duration of 1500 s). The total treatment duration was approximately 3000 s. Steam was continuously injected during the whole densification process at a maximum manometer pressure of 550 kPa under an increasing mechanical manometer platen pressure up to 6 MPa on the specimens. At the end of the treatment, steam injection was stopped and steam was purged through the holes in the platens. For densification without steam, the process parameters were kept the same but no steam was injected into the press.

### 2.2. Determination of Chemical Properties

#### 2.2.1. X-ray Photoelectron Spectroscopy

All of the resulting powder samples were sent to Université Laval (Laboratoire d’analyse de surface, Québec, Canada) to perform the surface element composition analysis using X-ray photoelectron spectroscopy (XPS, Kratos (Manchester, UK), Axis-Ultra). The emitted photoelectron take-off angle was at 30° with respect to the bottom of the cup. Base pressure in the analysis chamber was 5 × 10^−10^ Torr. A hybrid lens mode was applied. XPS spectra were recorded using a monochromatic AlKα source operating at 300 W with 20 eV pass energy and a step size of 0.05 eV. These high energy resolution spectra were used for chemical analysis.

#### 2.2.2. ATR-FTIR

THM-densified and control specimens (50 × 50 mm) were examined by attenuated total reflection Fourier transform infrared spectroscopy (ATR-FTIR) in a Nicolet OMNIC 560 Fourier Transform spectrometer. Specimens were scanned at between 4000 and 700 cm^−1^ with a resolution of 1 cm^−1^; the average of 64 scans was recorded.

#### 2.2.3. Py-GC/MS

Samples were pyrolyzed using pyrolysis gas chromatography (GC) (Varian CP 3800) mass spectrometry (MS) (Varian Saturn 2200 MS/MS, 30–650 amu). The carrier gas is helium with a flow rate of 1.0 mL/min. The GC injector temperature was set at 250 °C with a split ratio of 1:200. The GC oven was kept at 45 °C for 2 min and then heated to 270 °C at 5 °C/min; the final temperature was held for 5 min. The MS was operated in electron impact (EI) mode using an electron ionization energy of 70 eV and a mass range of 35–450 m/z was scanned in 36 s. All compounds in the obtained mass spectra were identified by referring to the National Institute Standards and Technology (NIST 2000) mass spectral libraries. The sum areas of all pyrolysis product peaks areas were normalized to 100%. Each compound concentration was calculated by the ratio of its peak area to the sum area in percent.

## 3. Results

### 3.1. XPS Analysis

**Figure 2:**
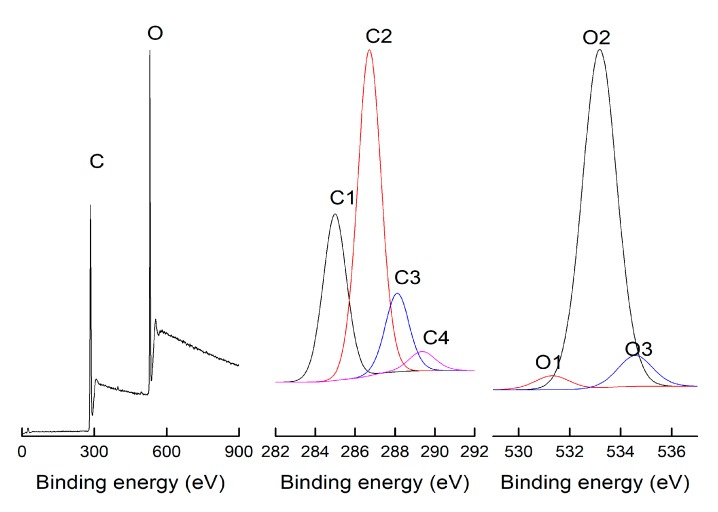
Typical X-ray photoelectron spectroscopic survey spectra of the control sugar maple wood.

Figure 2 presents a typical XPS survey spectra and high-resolution scans of C1s and O1s spectra with their decomposition into four and three components, respectively. The elemental composition of wood surface mainly includes carbon (C), hydrogen (H) and oxygen (O). All of the elements which are analyzed to a depth of about 10 nm are detectable by the XPS spectra, except H [19]. As shown in Figure 2, carbon and oxygen are two principle chemical elements on the wood surface; small amounts of other elements like nitrogen, calcium and sodium can also be found. To take a deeper insight into the surface chemical composition, the carbon bands (C1s) are interpreted as C–C or C–H (C1), C–O (C2), C=O (C3) and O=C–O (C4). The oxygen bands (O1s) are interpreted as C=O (O1), C–O (O2) and phenolic oxygen (O3).

Surface chemical elementary compositions (in %) and oxygen/carbon atomic (O/C) ratios of the studied samples are presented in Table 1. The sum of C1 and C2 constitute more than 80% of all the carbons on the surfaces of the five studied samples, whereas C3 and C4 were in the minority. Most of the C1 could be attributed to aliphatic and aromatic carbons in lignin and extractives [17,19]. Kocaefe et al. [20] reported that C1 corresponds to carbon in lignin, hemicelluloses and extractives [21]. The C2 can result from lignin, hemicelluloses and cellulose, but predominantly from cellulose as –CHOH [17]. Koubaa et al. [22] also proposed that the cellulose attributes a higher contribution to C2 than lignin. The C3 mainly results from hemicelluloses and cellulose, the C4 is associated with hemicelluloses and extractives [23]. At the same densification temperature, the concentrations of C1 and C4 of samples treated with steam were lower than those of samples treated without steam, whereas the concentrations of C2 and C3 showed an opposite tendency. Under the same steam conditions, the concentration of C1 decreased with increasing densification temperature. The C2 concentrations of samples treated with steam were higher than those of the control and samples treated without steam. The C2 mainly originates from cellulose. The increase of C2 concentration indicated that the relative content of cellulose increased after densification at 180 °C with steam and after 200 °C with and without steam. Belleville et al. [13] reported that the increase of the C2 component could be also associated with the formation of furanic compounds from xylan due to the degradation of hemicelluloses. O2 (C–O) corresponds to oxygen in cellulose and hemicelluloses [22,24]. The increase of O2 after densification might be explained by the same reasons as the increase of C2 (C–O).

The oxygen to carbon (O/C) atomic ratio is an important indication for the degradation of cellulosic materials and polymers, which is widely used in quantitative XPS analysis. In general, the theoretical O/C ratio of cellulose is 0.83, the hemicellulose (pentosans) has an O/C molar ratio of approximately 0.8 and the O/C ratio value of lignin is around 0.33 [21]. The extractives have the lowest O/C ratio of 0.1 [25]. Therefore, the O/C ratio can reflect the relative contents of cellulose, hemicelluloses and lignin. For example, a high content of carbohydrates on the wood surface should result in a high O/C ratio. Conversely, a low O/C ratio reflects the presence of more lignin and extractives on wood surface. In this study, the O/C ratio of all the five samples was below 0.5, which suggested that there was a relatively high proportion of the lignin and extractives on the sugar maple wood surface. Additionally, after the densification treatment, the O/C ratio of the samples slightly increased, except for the samples densified at 180 °C without steam (Table 1). Kamdem et al. [26] reported that the higher carbon content, especially the C1 component in the C1’s spectra, suggested the presence of carbon-rich substances, which could be mainly related to extractives. The decrease of that component was almost regular following the thermal treatment which could be related to the loss of some carbon-rich extractives. A slight increase in C1 content was found for samples densified at 180 °C without steam. This might indicate a larger amount of carbon-rich substances remaining on its surface, resulting also in a relatively lower O/C ratio. The slight increase of the O/C ratio following the thermal treatments might suggest an increase of chemical substances with oxygenated functionalities following the treatments with steam and/or high temperature (200 °C). It is interesting to note the regular increase of C4 following the thermal treatment under all conditions, which could indicate that the conditions applied were favorable to the introduction of carbon–oxygen groups (O=C–O) in wood components. The slight increase of the O/C ratio might also be due to the increase of the relative content of cellulose (the O/C ratio of cellulose is 0.83).

### 3.2. ATR-FTIR Analysis

**Figure 3:**
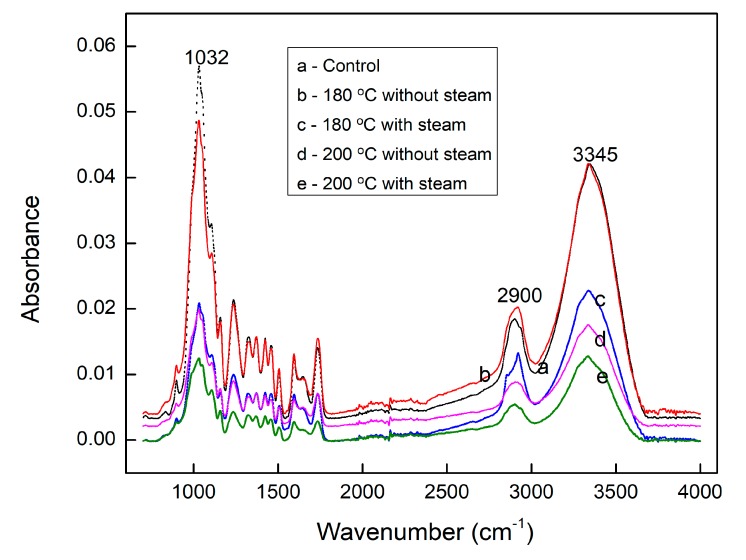
Typical ATR-FTIR spectra for the control and densified sugar maple wood samples.

**Figure 4:**
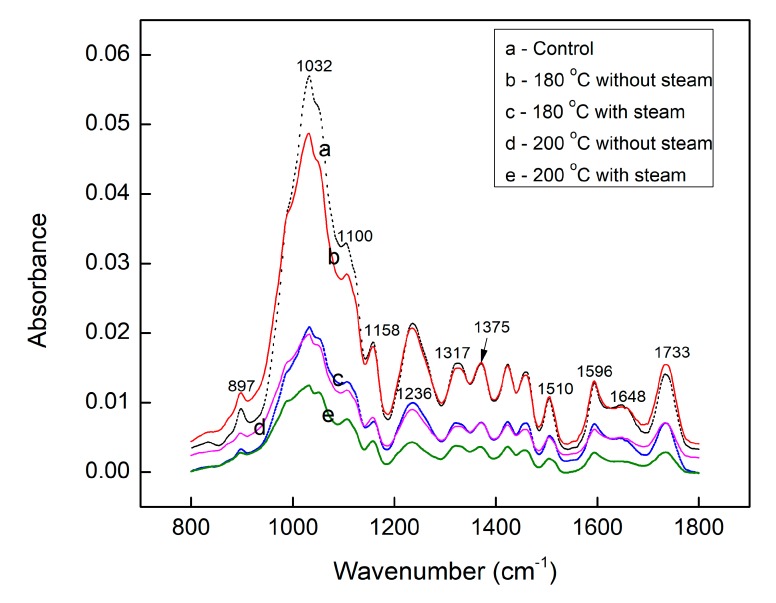
ATR-FTIR fingerprint region spectra of the control and densified sugar maple wood samples.

ATR-FTIR was used to analyze changes of the structures of the wood surface component (chemical functional groups) after the THM densification treatment. Figure 3 presents typical ATR-FTIR spectra for control and densified sugar maple wood samples. The whole spectra presented in Figure 3 can be generally divided into two regions: (1) The functional region located in the range of 2700–3800 cm^−1^, with two main absorption peaks at 2900 cm^−1^ and 3345 cm^−1^ corresponding to the absorption of C–H bond stretching and O–H bond stretching, respectively, and (2) the fingerprint region distributed in the range of 800–1800 cm^−1^, assigned to swing, stretching or bending vibrations of different functional groups of wood components [17]. Both lignin and carbohydrates, containing C–H and O–H functional groups, would have absorption bands appearing in this functional region. It is difficult to distinguish between the components which induced the variation of absorption bands in this region. Therefore, the attention was focused on the peaks in the fingerprint region. Well-defined ATR-FTIR fingerprint region spectra of the control and the samples densified under different conditions are presented in Figure 4. Compared to the functional region, the fingerprint region provides more information about the chemical changes of wood after THM densification.

The peak at 1032 cm^−1^ is assigned to holocellulose bonds. As shown in Figure 4, compared to the control sample, the absorption intensities of this peak for densified samples was much lower than that of the control sample. This might be due to the degradation of hemicelluloses that occurred during the densification treatment, resulting in a decrease in hemicellulose content. The absorption intensity of this peak for samples densified with steam was much lower than that of the samples densified without steam at the same temperature. This may indicate that more degradation of hemicelluloses occurred when the samples were densified with steam injection.

To evaluate and compare the relative content of different functional groups in the fingerprint region (Figure 4), peaks with maximum intensities at 1032 cm^−1^ were normalized to the same value and the results are presented in Table 2. A peak at 1733 cm^−1^ is usually assigned to C=O stretching in hemicelluloses, lignin and extractives. After THM densification at 180 °C with/without steam and 200 °C without steam, a slight increase in intensity of this peak (1733 cm^−1^) can be observed, which might be due to the increase in ester-like component and could be related to the increase of C4 component found by analysis of XPS spectra for the same surfaces. According to other published results [27,28,29], absorption peaks at 1510 cm^−1^, 1596 cm^−1^ and 1648 cm^−1^ are assigned to aromatic skeletal vibration in lignin. Higher intensities can be observed for samples densified at 180 °C with/without steam and 200 °C without steam compared to the control sample. This might be caused by the splitting of the aliphatic side chain in lignin and/or condensation reactions [30]. The absorption peaks at 1100 cm^−1^, 1158 cm^−1^ and 1375 cm^−1^ corresponded to the O–H bond vibration associated with cellulose, asymmetric stretching of the C–O–C bond and C–H deformation in carbonhydrates, respectively. The glycosidic cellulose bonds at 897 cm^-1^ was assigned to asymmetric out-of-phase ring stretching of the C1–O–C4 in cellulose [31,32]. The 1317 cm^−1^ band corresponded to –CH_2_ wagging in the crystalline cellulose [33,34]. Delmotte et al. [28] reported peaks at 897, 1317 and 1424 cm^−1^ attributed to –CH_2_ bending vibration in cellulose [17,35,36]. Higher intensities of these peaks (at 897 cm^−1^, 1317 cm^−1^ and 1424 cm^−1^) were observed for the samples examined after densification. This could indicate a relative increase in cellulose after densification.

This result can be related to the slight increase of O/C ratio, as determined by comparative XPS studies of the wood surfaces before and after the densification process discussed previously.

### 3.3. Py-GC/MS Analysis

**Figure 5:**
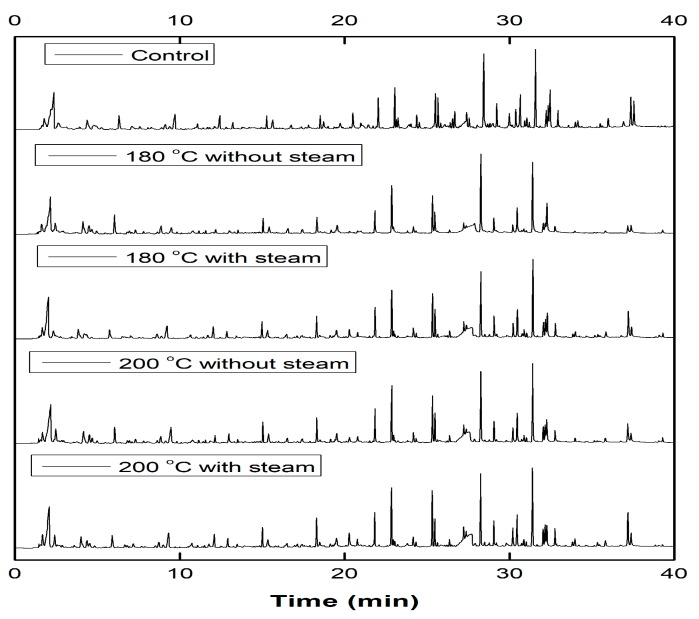
Pyrolysis chromatograms of the control and densified samples.

Py-GC/MS was used to investigate the structural changes of the wood surface following different THM densification treatments. Pyrolysis chromatograms of the control and densified samples are presented in Figure 5. Major compounds from the mass spectra, presented in Figure 5, were identified using the National Institute Standards and Technology (NIST) mass spectral libraries. The low retention time between 0 and 18 min corresponds to the pyrolysis products of carbohydrates, while the majority of the compounds corresponding to a retention time of 18 to 38 min are derived from lignin. The major pyrolysis products and their relative area percentages obtained from the Py-GC/MS analysis of the control and densified sugar maple wood are presented in Table 3.

The pyrolysis of hemicelluloses mainly produces ketones, acetic acid and furfural. Lignin produces most of the phenols and phenol derivatives. The pyrolysis of lignin mainly involves three processes: (1) Lignin depolymerization, (2) further transformation of depolymerization products into guaiacol or syringol derivatives and (3) further transformation of guaiacols or syringols into simple phenols and aromatics [37,38]. Cellulose is the dominant source for the production of anhydrous sugars and furans [39]. Cellulose first decomposes into oligosaccharides, then degrades into anhydrous monosaccharides, such as D-Allose and levoglucosan, these monosaccharides can further transform into furans or hydroxyacetaldehyde [39].

As shown in Table 3, the release of phenyl units with short chains (such as 2-methoxy-phenol, 2-methoxy-4-methyl-phenol and 2,6-dimethoxy-phenol) significantly increased for densified samples. These observations could indicate that some cleavage of the side chains in phenyl-propane units in sugar maple lignin occurred during densification treatment. The content of guaiacyl-type pyrolysis products (2-methoxy-phenol, 2-methoxy-4-methyl-phenol, 2-methoxy-4-vinylphenol and eugenol) obtained from densified samples was somewhat higher as compared to those obtained from the control sample. A slight increase in ketone contents (3-methyl-4-penten-2-one and 2-Cyclopentene-1,4-dione) released from the densified samples could be taken as an indication of some oxidation occurring during the densification process. The results of XPS analysis also revealed that the O/C ratio on wood surface slightly increased after densification.

Sugar maple lignin consists of high percentage of syringyl-type units [13,40] and the syringyl-type units seem to be the major source of lignin pyrolysis-derived compounds. As shown in Table 3, a large number of syringyl-type compounds were detected, such as 2,6-dimethoxy-phenol, 3,5-dimethoxyacetophenone, 4-allyl syringol, 4-hydroxy-3,5-dimethoxy-benzaldehyde, acetosyringone, trans-sinapaldehyde and 2-allyl-1,4-dimethoxy-3-methyl-benzene. Particularly, 4-allyl syringol was detected as one of the main syringyl-type compounds. The content of 4-allyl syringol obtained from the densified samples was higher than that obtained from the control sample. Belleville et al. [13] suggested that phenol compounds were produced by lignin depolymerization through the cleavage of ether bonds. General oxidation reactions that could have occurred in syringyl units during densification process could lead to the formation of aldehyde-type and ketone-type compounds, as the concentrations of such compounds released from the densified wood are higher than those from the control sample, as can be observed by comparisons between the samples for the compounds 3,5-dimethoxyacetophenone and trans-sinapaldehyde.

The levoglucosan content obtained from the control and the samples densified at 200 °C with steam increased significantly from 0.57% to 4.8%. This could be taken as an indication that the relative content of cellulose increased after densification treatments. This result would be in agreement with the results of XPS and FTIR analysis discussed previously. Another interesting finding was that both the quantities of carbohydrate-derived and lignin-derived compounds obtained by pyrolysis from the densified samples were significantly higher than those obtained from the control sample. Lignin-derived and carbohydrate-derived compound ratios decreased after the densification treatment. This result is also in agreement with our finding by XPS regarding the increase in O/C ratio, as this ratio does increase when the ratio of lignin/carbohydrates derived components decreases, which is the result of Py-GC/MS presented in Table 3.

According to the above analysis, it can be concluded that the densification treatment favored the depolymerization of hemicelluloses and cellulose, resulting in increased anhydrous sugar (levoglucosan) release during the pyrolysis process. Densification also facilitated the cleavage of lignin side chains, as indicated by increased phenyl units with short chains released during the pyrolysis process.

## 4. Conclusions

THM densification treatment resulted in significant chemical changes on the wood surface. The results of the ATR-FTIR spectra confirmed the decomposition of hemicelluloses and the increase of the relative cellulose and lignin content on the wood surface. The Py-GC/MS and XPS results seem to indicate an increase in the O/C ratio in the densified wood samples. This shows that chemical substances containing oxygenated functionality were formed after densification. The densification treatment promoted the depolymerization of hemicelluloses and cellulose, resulting in an increase in the anhydrous sugars (levoglucosan) released during the pyrolysis process. Densification also facilitated the cleavage of lignin side chains, resulting in increased phenyl units with short chains released during the pyrolysis process. After densification treatment, the increase of the relative cellulose content should lead to the improvement of the mechanical properties of wood. More degradation of hemicelluloses occurred for samples densified with steam, resulting in better dimensional stability of wood compared to the samples densified without steam.

## Figures and Tables

**Table 1:** Surface chemical element composition and oxygen/carbon atomic ratio of the studied samples.

Source		Control	180 °C without Steam	180 °C with Steam	200 °C without Steam	200 °C with Steam
Elements (%)	C	67.96	68.63	66.16	66.86	66.75
	O	31.50	30.79	33.37	32.59	32.98
	Others	0.54	0.58	0.47	0.55	0.27
C1s component (%)	C1	28.11	32.83	26.02	25.68	25.12
	C2	55.06	51.92	57.27	56.56	57.01
	C3	13.43	10.91	12.88	12.51	13.81
	C4	3.40	4.34	3.83	5.25	4.06
O1s component (%)	O1	5.43	2.86	1.19	1.45	1.78
	O2	86.64	90.08	93.16	91.65	91.63
	O3	7.93	7.06	5.65	6.9	6.60
Atomic Ratio	O/C	0.46	0.45	0.50	0.49	0.49

**Table 2:** Relative intensities of peaks after normalization.

Peak Position (cm^−1^)	Peak Assignment	Peak Intensity after Normalization				
		Control	180 °C without Steam	180 °C with Steam	200 °C without Steam	200 °C with Steam
897	C1–O–C4 in cellulose	0.0091	0.0133	0.0091	0.0161	0.0127
1032	Holocellulose bonds	0.0570	0.0570	0.0570	0.0570	0.0570
1100	O–H bond in cellulose	0.0326	0.0328	0.0348	0.0331	0.0336
1158	C–O–C bond in carbonhydrates	0.0187	0.0211	0.0198	0.0225	0.0204
1236	C–O stretching in lignin and xylan	0.0214	0.0243	0.0272	0.0258	0.0196
1317	–CH_2_ wagging in the crystalline cellulose	0.0153	0.0170	0.0191	0.0185	0.0170
1375	C–H deformation in carbonhydrates	0.0153	0.0181	0.0192	0.0200	0.0159
1424	–CH_2_ bending vibration in cellulose	0.0155	0.0179	0.0198	0.0195	0.0167
1446	Aromatic C–H in lignin	0.0144	0.0139	0.0192	0.0176	0.0143
1510	Aromatic skeletal vibration in lignin	0.0094	0.0116	0.0138	0.0136	0.0083
1596		0.0126	0.0151	0.0188	0.0175	0.0126
1648		0.0097	0.0110	0.0132	0.0144	0.0072
1733	C=O stretching in hemicelluloses, lignin and extractives	0.0141	0.0181	0.0193	0.0204	0.0130

**Table 3:** Percentages of lignin and carbohydrate-related products released from the control and densified sugar maple wood.

Compounds	Origin	Treatments				
		Control	180 °C without Steam	180 °C with Steam	200 °C without Steam	200 °C with Steam
Cellulose/hemicellulose-derived compounds (peak area %)						
Dihydro-4-hydroxy-2(3H)-furanone	H	1.13	2.37	0.60	1.10	0.79
Propylene carbonate	H	0.65	1.23	0.52	0.82	0.64
(S)-5-hydromethyl-2(5H)-Furanone	H	0.22	0.41	0.24	0.24	0.20
Furfural	H	1.13	0.13	1.22	1.44	1.09
2-Furanmethanol	H	0.84	0.26	0.65	0.24	0.58
3-methyl-4-penten-2-one	H	0.29	0.57	0.48	-	0.43
2-Cyclopentene-1,4-dione	H	0.16	0.27	0.43	0.20	0.16
2(5H)-Furanone	H	0.55	1.16	0.66	0.74	0.56
5-methyl-2(5H)-Furanone	H	0.06	0.19	-	0.14	0.07
3-methyl-2,4(3H,5H)-furandione	H	0.38	0.74	0.47	0.51	0.32
Levoglucosan	C	0.57	-	2.31	2.43	4.8
5-hydroxymethyl-2-furancarboxaldehyde	C	0.53	1.25	1.18	0.93	0.87
Lignin-derived compounds (peak area %)						
Phenol	L	0.15	0.29	0.12	0.15	0.11
2-methoxy-phenol	L	0.08	1.27	1.12	1.22	0.99
2-methoxy-4-methyl-phenol	L	0.71	1.61	1.42	1.47	1.56
1,2-benzenediol	L	0.63	-	0.40	0.39	0.47
3-methoxy-1,2-benzenediol	L	1.17	-	0.96	0.68	1.26
4-ethyl-2-methoxy-phenol	L	0.50	0.29	0.49	0.71	0.56
2-methoxy-4-vinylphenol	L	1.61	2.21	1.75	2.26	1.62
2,6-dimethoxy-phenol	L	2.44	4.13	3.30	3.51	2.96
2-methoxy-3-(2-propenyl)-phenol	L	0.42	0.52	0.43	0.43	-
Eugenol	L	0.09	0.29	1.51	0.24	0.35
2-methoxy-4-(1-propenyl)-phenol	L	0.41	0.33	0.29	0.29	0.27
isoeugenol	L	1.40	1.73	0.72	1.57	1.23
1-(4-hydroxy-3-methoxyphenyl)-ethanone	L	0.57	0.26	0.50	0.48	0.40
3,5-dimethoxyacetophenone	L	4.83	6.07	4.93	5.38	4.15
4-((1E)-3-hydroxy-1-propenyl)-2-methoxy phenol	L	0.16	0.08	0.16	0.08	0.17
4-hydroxy-3,5-dimethoxy -benzaldehyde	L	2.03	2.61	2.17	2.10	1.80
4-allyl syringol	L	6.23	7.69	7.69	7.04	6.48
Acetosyringone	L	1.46	1.79	1.62	1.13	1.49
Trans-sinapaldehyde	L	1.91	0.87	2.18	1.52	2.14
2-allyl-1,4-dimethoxy-3-methyl-benzene	L	2.57	1.82	1.98	1.51	2.27
Carbohydrate-derived compounds		6.51	8.58	8.76	8.79	10.51
Lignin-derived compounds		29.37	33.86	33.74	32.16	30.28
Lignin-derived/carbohydrate-derived compounds		4.51	3.94	3.85	3.67	2.88

H: Hemicelluloses; C: Cellulose; L: Lignin; -: not detected.
